# A stab in the dark: chick killing by brood parasitic honeyguides

**DOI:** 10.1098/rsbl.2011.0739

**Published:** 2011-09-07

**Authors:** Claire N. Spottiswoode, Jeroen Koorevaar

**Affiliations:** 1 Department of Zoology, University of Cambridge, Downing Street, Cambridge CB2 3EJ, UK; 2 Percy FitzPatrick Institute, DST/NRF Centre of Excellence, University of Cape Town, Rondebosch 7701, South Africa; 3 Adviesbureau E.C.O. Logisch, Postbus 38, 2910 AA Nieuwerkerk aan den Ijssel, The Netherlands

**Keywords:** avian brood parasitism, virulence, chick killing, trade-offs

## Abstract

The most virulent avian brood parasites obligately kill host young soon after hatching, thus ensuring their monopoly of host parental care. While the host eviction behaviour of cuckoos (Cuculidae) is well documented, the host killing behaviour of honeyguide (Indicatoridae) chicks has been witnessed only once, 60 years ago, and never *in situ* in host nests. Here, we report from the Afrotropical greater honeyguide the first detailed observations of honeyguides killing host chicks with their specially adapted bill hooks, based on repeated video recordings (available in the electronic supplementary material). Adult greater honeyguides puncture host eggs when they lay their own, but in about half of host nests at least one host egg survived, precipitating chick killing by the honeyguide hatchling. Hosts always hatched after honeyguide chicks, and were killed within hours. Despite being blind and in total darkness, honeyguides attacked host young with sustained biting, grasping and shaking motions. Attack time of 1–5 min was sufficient to cause host death, which took from 9 min to over 7 h from first attack. Honeyguides also bit unhatched eggs and human hands, but only rarely bit the host parents feeding them.

## 1. Introduction

Avian brood parasites vary greatly in virulence among species [1]. While many brood parasites are raised alongside host chicks and can be relatively benign, three independently evolved brood parasitic groups have evolved extremely high degrees of virulence: the young parasite actively kills its foster siblings, thus ensuring that it monopolises the parental care provided by the host parents. First, in a clade of Old World cuckoos, including the well-studied common cuckoo *Cuculus canorus*, the parasitic chick hoists host eggs or young onto its back and tips them out of the nest [2]. Second and third, in the striped cuckoo *Tapera naevia* of the Neotropics [3] and the honeyguides (Indicatoridae) of Africa and Asia [4], the parasitic chick kills host young by attacking them with specially modified bill hooks. Chick killing has never been witnessed in the striped cuckoo, but rather surmised from the presence of bill hooks and dead host young [3]. The reproductive biology of honeyguides has also remained very poorly known, despite these intriguing morphological and behavioural adaptations that are absent in their non-parasitic relatives, the woodpeckers (Picidae) and barbets (Capitonidae) [4] (although a single maxillary hook is sometimes used in intraspecific sibling aggression in at least one species of bee-eater (Meropidae) and kingfisher (Halcyonidae) [5]).

Nestlings of only five of the 17 honeyguide species have ever been observed, but since all including the basal genus *Prodotiscus* show bill hooks and host young have never been found alongside them [6], it is assumed that all are chick-killing brood parasites. To our knowledge, the only description to date of honeyguide killing behaviour is that of Gordon Ranger in 1952, describing two chicks in his outstretched hand: he reported that the honeyguide bit the host in ‘great grasping bites’, and that in testing the honeyguide's biting power he ‘had his tongue punctured by the upper hook’ [7]. However, no reports exist under natural conditions in the nest, and the behaviour has never been filmed nor documented in detail.

The Afrotropical greater honeyguide *Indicator indicator* is remarkable for its interactions with other species. It shows a unique mutualism with human honey-gatherers whom it guides to bees' nests [8], as well as being a highly virulent brood parasite of hole-nesting birds. Here, we report on killing behaviour by the greater honeyguide in host nests in Zambia, aiming to document for the first time (i) how killing behaviour contributes to parasitic virulence, given that laying females of this species also puncture host eggs [9]; (ii) the conditions under which killing behaviour occurs, including timing during development and size disparity between the parties; and (iii) the nature of the killing behaviour itself.

## 2. Material and methods

We conducted fieldwork in the Choma region of southern Zambia during September–November 2008–2010, in a *ca* 5000 ha area centred on 16°45′′ S, 26°54′′ E. The habitat is mixed miombo woodland, seasonally flooded depressions and tobacco cultivation. Here, the little bee-eater *Merops pusillus* [9] is the commonest host, breeding in subterranean burrows (*ca* 0.5 m long and 0.2–0.5 m underground) typically dug into the roofs of aardvark (*Orycteropus afer*) holes, or sometimes earth banks or bushpig (*Potamochoerus larvatus*) diggings. Clutch size is three to six eggs.

We filmed chick behaviour by inserting an infrared camera at the end of the bee-eaters' access tunnels. Cables led to the surface where a digital recorder was concealed nearby. Bee-eaters ignored this equipment. Killing behaviour was filmed at five nests of three host species (little bee-eater three, swallow-tailed bee-eater one and African hoopoe one; the last breeds in tree holes), involving four honeyguide chicks and six host chicks. In two nests, we had moved the honeyguide from another nest, matched in developmental stage, but attacked by ants or predators; foster nests are excluded from mass analyses. Data on honeyguides' impact on hosts all refer to the commonest host species, the little bee-eater.

## 3. Results

Of 172 little bee-eater nests followed, 113 (65.7%; varying from 56.5 to 70.1% among years) were visited by a honeyguide, as revealed by a honeyguide egg and/or punctured host eggs. However, of these visited nests, 64 (56.6%) were deserted by hosts at this early stage. Therefore, a parasitic egg was incubated by hosts in only 49 (28.5%) of all host nests. Honeyguide oviposition occurred any time from the start of host egg-laying to late into the host incubation period. Greater honeyguides did not remove host eggs, but (uniquely among honeyguides [6]) punctured them at the time of laying. Embryos in punctured eggs usually died [9], but sometimes remained viable. Moreover, host eggs were sometimes missed or laid subsequent to the honeyguide's visit. Thus, only 67 per cent (*n* = 194) of host eggs in parasitised nests were punctured. In 30 of 55 parasitised nests (55%), at least one (range one to four) host egg was unpunctured and could have hatched; proportions were similar for other host species. Hence, egg puncturing only partially removed the scope for subsequent chick killing.

**Figure 1.**
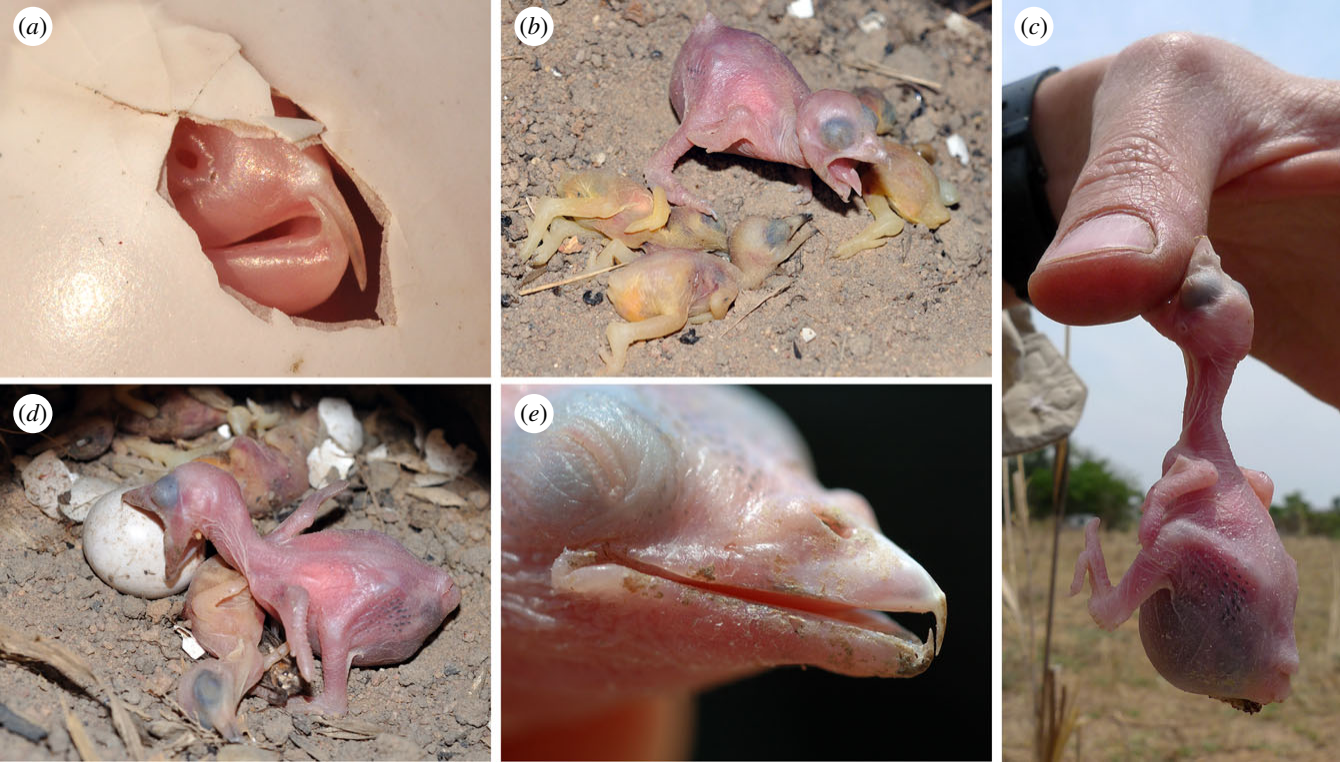
(*a*) Hatching lesser honeyguide, showing fully developed bill hooks; (*b*) greater honeyguide chick with three recently killed little bee-eater hatchlings; (*c*) biting human hand; (*d*) biting unhatched swallow-tailed bee-eater egg; (*e*) aged about 8 days. All photos are from different nests.

Honeyguides always hatched before host young, despite sometimes asynchronous laying (late-laying females puncture host eggs more extensively [9]). Honeyguide eggs took 15–17 days (*n* = 10; in one case 19 days) to hatch, compared with 18–20 days for hosts [10]. Hosts hatched asynchronously with respect both to honeyguides and to one another. Without exception, the honeyguide was several (*ca* 2–4) days old when the first host chick hatched. At hatching, honeyguides weighed *ca* 3 g, possessed fully developed bill hooks (figure 1*a*) and immediately attempted to bite our fingers when handled. Honeyguides had attained substantially greater weight by the time host chicks hatched and were killed: 6.06–11.08 g (mean = 9.11, *n* = 4) for the first-hatched host and 10.22–13.29 g (mean = 11.29, *n* = 3) for the last-hatched. Hosts were attacked soon after hatching (17, 37 and 50 min in the three cases accurately recorded), when little bee-eaters weighed less than 2 g (1.79 g, range 1.50–1.92, *n* = 4).

**Figure 2.**
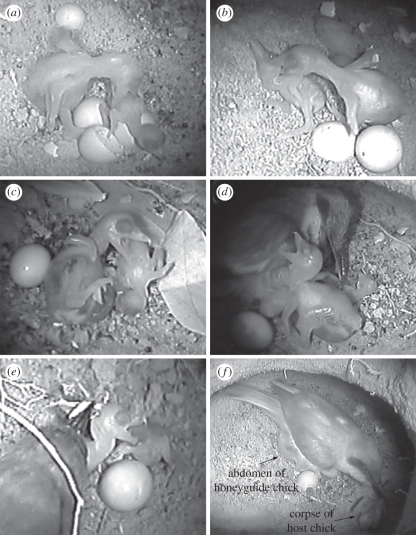
Stills from infrared footage within host nest chambers: (*a*–*c*) greater honeyguide chicks attacking newly-hatched little (*a*,*c*) and swallow-tailed (*b*) bee-eaters; (*d*) greater honeyguide biting host parent; (*e*) host parent attempting to feed greater honeyguide busy attacking its own chick; (*f*) little bee-eater parent removing its dead chick while brooding a greater honeyguide (distended honeyguide abdomen visible below bee-eater).

Honeyguide chicks attacked host chicks using their bills (figure 2*a*–*c* and electronic supplementary material, videos S1–S4). They typically held onto hosts for a period of time (mean bout length 17 s, range 1–249 s; *n* = 60 bouts) during which they repeatedly opened and closed their mandibles, and often shook the host chick, either from side to side or up and down by rocking on their hindlegs (figure 2*a*–*c* and electronic supplementary material, videos S1–S4). Honeyguides reached out haphazardly rather than targeting particular body regions. They most commonly bit the back (*n* = 20 bites), head (14) and abdomen (14), and occasionally the neck (5), wings (4) and legs (3). Biting rarely caused open wounds, but rather haemorrhaging underneath the skin and heavy bruising (figure 2*b*). As previously noted [7], bite strength was considerable when felt on the human hand (figure 2*c*). Host chicks sustained an average of 177 s of direct attacks (range 55–308 s, *n* = 5 chicks), but from the time of first attack, hosts took from 9 min to over 7 h to die. Hosts did not attempt to avoid attacks and after a first bout of attack soon ceased any attempts to beg for food. Host corpses usually remained and decomposed in the nest, but in one nest a parent removed two of three corpses (figure 2*f*).

Honeyguides occasionally bit unhatched host eggs, but failed to puncture their shells (figure 1*d*). Only once did a honeyguide bite a host parent (figure 2*d*), despite being frequently brooded by them. A host chick was once attacked while host parents attended the nest (figure 2*e*).

The bill hook is retained for at least 14 days (figure 1*e*), corresponding to potentially late host encounters in large and asynchronously hatching host clutches. Hooks do not appear to be shed (cf. [4]), but rather grow gradually into the growing bill and are no longer detectable *ca* one week before fledging (which occurs at about 30 days).

## 4. Discussion

Virulence in the greater honeyguide results from a succession of three specialised adaptations which together ensure the death of all the host young: (i) egg puncturing by the laying female [9], which we show to eliminate two-thirds of host eggs; (ii) internal incubation by the laying female for an additional 24 h prior to egg-laying [11], that in combination with apparently rapid embryonic development [6], results in honeyguides invariably hatching ahead of surviving hosts; and (iii) highly effective killing behaviour by honeyguide hatchlings, which we show to ensure host death within hours of their hatching.

Host killing is carried out by blind hatchlings in total darkness, and the proximal cues used by honeyguides to start and stop biting remain unclear. Honeyguides sometimes lay alongside host young for tens of minutes before suddenly starting to attack, locating the host chick by repeatedly biting into mid-air while rapidly moving the head from side to side. Attacks often ceased when host chicks lay maimed and largely immobile, suggesting that movement may be a more important cue for attack than body heat. We only once witnessed a honeyguide biting a host parent, in spite of regularly being brooded by them; perhaps either the parents' food calls, or their feathered texture, serve as cues to prevent honeyguides from biting the hand that feeds them.

The degree of virulence shown by brood parasites should be determined by a trade-off between the costs and benefits of chick-killing, just as pathogen virulence is shaped by trade-offs [1]. What are the potential costs of chick-killing by honeyguides? First, killing behaviour itself might impose energetic costs. Eviction behaviour entails growth costs to common cuckoo chicks, albeit recoverable ones (e.g. [12]). In greater honeyguides, 1–5 min of active biting behaviour sufficed to kill host young, although visible deep breathing and long periods of inactivity often followed attacks (electronic supplementary material, videos S1 and S2). Experiments are needed to assess the energetic costs of this physical exertion. Moreover, morphological adaptations to chick killing may entail costs. Aside from bill hooks, honeyguides′ powerful jaw motion is likely to involve adaptive modifications to musculature, as might their shaking movements (electronic supplementary material, videos S3 and S4); these may require significant energetic investment [13].

Second, the fitness costs versus benefits of honeyguide chicks′ sole occupancy of the nest remain to be quantified. When parasitizing host species that they are easily able to outcompete in sibling competition, such as little bee-eaters, parasites may do better to reduce virulence and co-opt host nestling begging to their own advantage [14]. We might speculate that honeyguides are trapped in an ancestral strategy of extreme virulence that prevents them from exploiting such adaptive opportunities.
